# Injection Molding of Encapsulated Diffractive Optical Elements

**DOI:** 10.3390/mi14061223

**Published:** 2023-06-09

**Authors:** Stefan Wagner, Kevin Treptow, Sascha Weser, Marc Drexler, Serhat Sahakalkan, Wolfgang Eberhardt, Thomas Guenther, Christof Pruss, Alois Herkommer, André Zimmermann

**Affiliations:** 1 Hahn-Schickard, Allmandring 9B, 70569 Stuttgart, Germany; sascha.weser@hahn-schickard.de (S.W.); marc.drexler@hahn-schickard.de (M.D.); serhat.sahakalkan@hahn-schickard.de (S.S.); wolfgang.eberhardt@hahn-schickard.de (W.E.); thomas.guenther@ifm.uni-stuttgart.de (T.G.); andre.zimmermann@ifm.uni-stuttgart.de (A.Z.); 2 Institute for Micro Integration (IFM), Faculty 7—Engineering Design, Production Engineering and Automotive Engineering, University of Stuttgart, Allmandring 9B, 70569 Stuttgart, Germany; treptow@ito.uni-stuttgart.de; 3 Institute for Applied Optics, Pfaffenwaldring 9, 70569 Stuttgart, Germany; pruss@ito.uni-stuttgart.de (C.P.); herkommer@ito.uni-stuttgart.de (A.H.)

**Keywords:** ultra-precision milling, laser direct writing, micro lens array, molding, optical elements, micro manufacturing, micro replication

## Abstract

Microstructuring techniques, such as laser direct writing, enable the integration of microstructures into conventional polymer lens systems and may be used to generate advanced functionality. Hybrid polymer lenses combining multiple functions such as diffraction and refraction in a single component become possible. In this paper, a process chain to enable encapsulated and aligned optical systems with advanced functionality in a cost-efficient way is presented. Within a surface diameter of 30 mm, diffractive optical microstructures are integrated in an optical system based on two conventional polymer lenses. To ensure precise alignment between the lens surfaces and the microstructure, resist-coated ultra-precision-turned brass substrates are structured via laser direct writing, and the resulting master structures with a height of less than 0.002 mm are replicated into metallic nickel plates via electroforming. The functionality of the lens system is demonstrated through the production of a zero refractive element. This approach provides a cost-efficient and highly accurate method for producing complicated optical systems with integrated alignment and advanced functionality.

## 1. Introduction

Optical systems play a crucial role in everyday life and industry as they are utilized in various applications, including cell phone cameras [1], driver assistance systems [2], inspection systems [3], or sensor systems [4]. Due to the advances in miniaturization and integration, optics become increasingly small and inconspicuous. In particular, components such as diffractive optical elements (DOE) add a significant value to these advances. They reduce the number of optical elements in an optical system while improving their functionality, thus enabling compact and lightweight setups.

**Figure 1:**
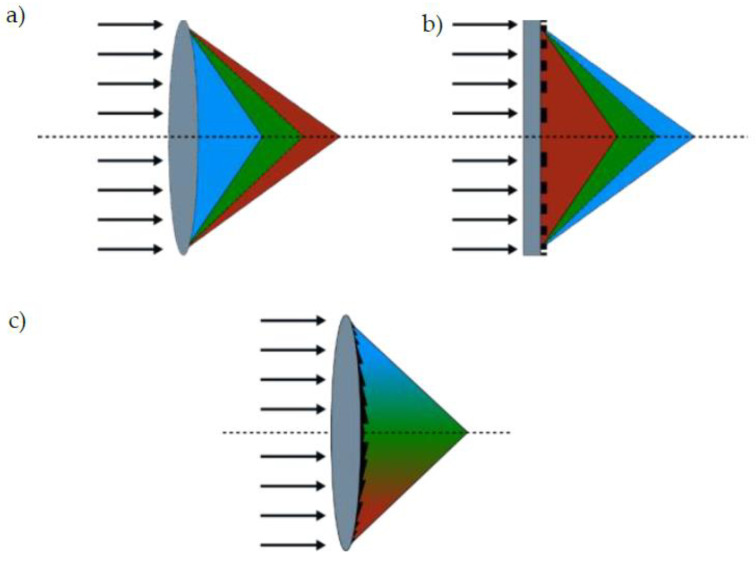
Influence of lens forms and structures on broadband light; (**a**) refraction of broadband light passing a conventional bi-convex lens; (**b**) diffraction based on surface microstructures; (**c**) combination of both effects acts complementary.

So-called hybrid optics are based on the combination of both conventional refraction and diffraction. Both phenomena are wavelength-dependent and suffer from chromatic aberration. Dependent on material properties and wavelength, broadband light is chromatically split when focused through conventional or diffractive lenses (Figure 1a,b, respectively), resulting in different focal lengths for different wavelengths, whereas the shorter wavelengths are stronger refracted through a conventional lens, meaning they are less diffracted through diffractive lenses.

Chromatic splitting in refraction and diffraction acts in opposite directions. A diffractive element, therefore, may be used to compensate the chromatic aberrations of a refractive lens as shown in Figure 1c and by further sources [4,5,6]. Combining the chromatic splitting of refractive and diffractive elements enables optical corrections to occur in small spaces [7,8]. In the past, numerous application examples for hybrid optics have been demonstrated, such as hybrid micro lens arrays [9], ultrathin endoscope objectives [10], or fiber-optical trapping [11]. Common to all of them are extremely close tolerances and high effort required for production and alignment in between production steps as well during assembly in a multi-optical system. Therefore, optical applications are often placed in the high-priced range.

Due to cost-efficient production, polymer optics have become the components of choice compared to regular glass optics in recent years when serving consumer markets and the automotive [12] and medical sectors [9]. While glass processing has regained a lot of attention in recent years [13,14], injection compression molding [15,16] and hot embossing [17] are powerful mass production tools available to produce high numbers of low-cost polymer lenses with high reproducibility. Abrasive methods such as ultra-precision milling [17] and turning [18] were proven to be valid fabrication methods for lenses with low surface roughness in the range of Ra < 10 nm. In the past, numerous approaches were shown to add diffractive structures to the lens surface via precision milling. Examples include fast tool servo-assisted milling [19] or diamond tooling [20]. These techniques were used to produce single lenses in glass or for tool inserts, but they lack flexibility. They are also limited due to the milling tool radius as well as the choice of material.

Additive manufacturing techniques such as digital light processing (DLP), grey-scale DLP [21,22], and direct laser writing [23] enable small batch production with micro and nanometer features on the lens surface, thus increasing the possible use cases with further integrated functions, but they also have disadvantages such as complexity and a high cost. The production of diffractive lenses with micro features generated via focused ion beam [24] or two-photon lithography [25,26] reaching the top end in terms of accuracy for processing optical features has been shown already. Most of these methods ensure a high accuracy, but until now, curved and reflective surfaces have posed a challenge for many of the techniques mentioned. In particular, the production of diffractive structures on curved surfaces has proved to be extremely complicated, but this has been demonstrated using lithography and laser-based writing [27,28]. On the other hand, numerous new applications in the consumer market have increased the need for complex low-cost optical components.

In this work, we present a process chain to produce high-precision diffractive structures on curved surfaces via the use of laser direct writing, electroformed with nickel, producing high-quality molding tool inserts. These inserts can be used for the high-accuracy injection compression molding of refractive polymer lenses with diffractive surface structures.

One major disadvantage of microstructured polymer optics compared to glass optics is their sensitivity against pollution or mechanical influences such as scratches. Additionally, the in-process alignment of several optical components often leads to extremely high tolerance costs that originate from the need for clean adjustment processes as well as high-accuracy optic mounts. This paper proposes additional adjustment structures integrated in the lenses for the manufacturing of encapsulated and robust microstructured polymer lenses.

## 2. Materials and Methods

**Figure 2:**
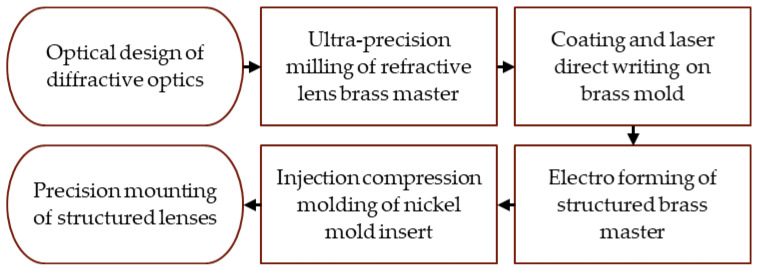
Process chart for manufacturing microstructured polymer lenses.

The process chain involves the ultra-precision turning of a brass substrate followed by spin coating of photoresist and laser direct writing of diffractive microstructures on the curved substrate. Afterwards, the mold insert for injection molding was fabricated using electroforming of the substrate. Diffractive structures and a refractive master substrate were aligned during the structuring process while additional passive alignment structures were included in the mold insert for encapsulation and alignment to additional lenses. The polymeric lens system was designed as a zero refractive element due to the combination of diffractive structures and the refractive surface shape. In combination with a second polymer lens, an encapsulated zero refractive element is presented. The polymer lenses were produced via injection compression molding and adjusted afterwards. A flow chart representing the process chain of the study is shown in Figure 2.

### 2.1. Ultra-Precision Turning of the Master Structures

**Figure 3:**
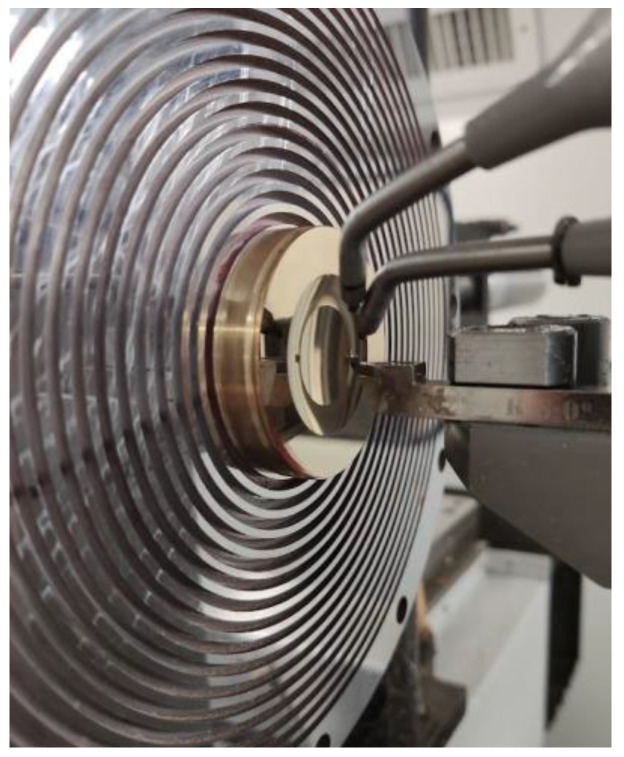
Turning of the brass master with optical surface finish.

The curved master substrates were fabricated via ultra-precision turning on a Precitech Ametek Freeform 700A machine (Ametek Precitech Inc., Keene, NH, USA). A surface roughness of R_a_ < 10 nm was achieved via diamond tooling using a diamond lathe tool with a cutting edge radius of r = 0.0705 mm from DiaTec GmbH (Pforzheim, Germany). The brass master is shown in Figure 3.

The tooling data used to process the final brass masters are shown in Table 1. The data used to process the final brass masters were experimentally determined, including the cutting speed, feed rates, and coolant types, by systematic adjustments and measurements to achieve optimal results.

### 2.2. Laser Direct Writing of Metallic Master Substrates

The laser direct writing machine, designed and manufactured by the institute for applied optics (ITO), enables the structuring of rotationally symmetric mold inserts made of glass or metal via scanning beam interference lithography (SBIL) [23].

**Figure 4:**
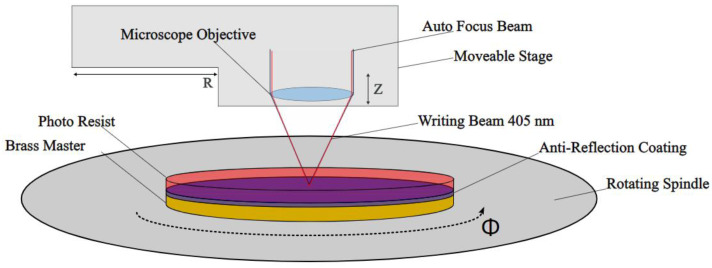
Illustration of the laser writing process for cylindrical substrates.

The experimental laser system is based on a Toptica BlueMode 05001 Laser (TOPTICA Photonics AG, Munich, Germany), operating at a wavelength of 405 nm. The machine is maintained in single-mode operation with an output power of 1 mW. Additionally, the laser exhibits a coherence length exceeding 25 m, providing the necessary characteristics for precise and controlled structuring of the photosensitive polymer. For this purpose, the inserts are spin-coated with a photosensitive polymer resist. Afterwards, the mold insert is fixed in the middle of a rotational base plate and the laser beam is focused onto the resin surface. The tunable z-position of the laser beam allows the resin to be hardened at different heights while the mold insert rotates, enabling the direct writing of diffractive gratings or Fresnel-like structures, as shown in Figure 4.

This setup permits grey tone lithography with up to 256 levels and works with slope angles of up to 15°. The machine is able to write structures below feature sizes of 500 nm on substrates with a diameter smaller than 300 mm. The writing process is followed by development and baking steps to harden the final structure.

Table 2 contains the spin coating parameters used for the anti-reflection coating AZ^®^ BARLi^®^-II solved in AZ^®^ EBR (Merck Performance Materials GmbH, Wiesbaden, Germany) solvent in a 30/70 ratio. A soft bake was performed afterwards for 3 min at 200 °C. The data in Table 2 and Table 3 were determined experimentally.

Table 3 contains the spin coating parameters for the actual photoresist layer. MICROPOSIT™ S1818™ from micro resist technology GmbH (Berlin, Germany).was used as the resist layer. A hard bake was performed at 90 °C for 45 min before structuring.

### 2.3. Electroforming of Structured Master Substrates

After development, the substrate was electroformed at Hahn–Schickard Stuttgart. In order to create a conducting layer, a 20 nm thin gold film was deposited via physical vapor deposition (PVD) on the initially insulating resist surface. Afterwards, a layer of around 4.5 mm of nickel was deposited via electroforming. Lectro-Nic HAR-1000 (MacDermid Enthone Inc., Langenfeld, Germany) electrolyte was used for the electroforming process. After the mechanical separation of the substrate, additional cleaning steps were performed in order to remove residual resist material. The nickel mold was cleaned in an ultrasonic bath in AZ^®^100 (Merck Performance Materials GmbH, Wiesbaden, Germany) remover for 60 min at 65 °C.

### 2.4. Injection Compression Molding with Structured Mold Inserts

Via electrical discharge machining, the nickel molds were cut to fit as a mold insert for an injection compression molding tool. Injection compression molding was performed on an Arburg Allrounder 370A (Arburg GmbH + Co KG, Lossburg, Germany) machine. Trogamid myCX (Evonik Industries AG, Essen, Germany)was used as the thermoplastic material with optical quality. Compression molding was performed using a movable compression stamper which was fly-cut to generate an optical flat surface. The tool insert on the opposite side was fixed using a mounting plate. During the injection compression molding process, the tool cavity was filled with polymer while the tool was not closed fully. After the injection of the polymer melt, the tool was closed fully in order to generate homogeneous pressure on the surface of the polymer part.

### 2.5. Alignment of Polymer Lens Components

**Figure 5:**
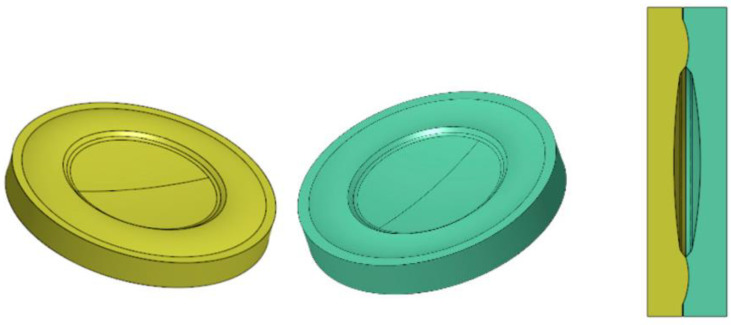
Radii on the lens surface used for joining.

One of the major challenges in polymer lens production is the relative alignment of the lenses to each other. To overcome this problem, the integration of additional mechanical structures is proposed, which enables the self-alignment of the lenses within the required precision for the given optical functionality. For this purpose, complementary curvatures are integrated in the outer frame of neighboring lenses. This allows the lenses to be centered when the curvatures are fitted onto each other, as shown in Figure 5.

The assembly of the lenses takes place in an assembly tool, which is especially constructed for this purpose. After the injection molding, both lenses are clamped into the tool and aligned and bonded simultaneously. A small bonding gap on the outer edge of the lenses allows an adhesive to be dispensed while the curvatures hinder the intrusion of the adhesive to the inner optical surfaces and prevent contamination.

**Figure 6:**
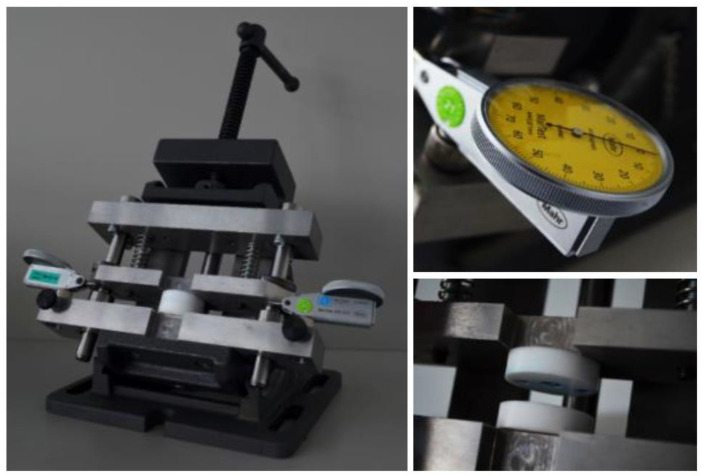
Assembly tool for lens adjustment and alignment.

The apparatus shown in Figure 6 contains two spring-loaded steel plates supported on two axes. These can be moved relative to each other. Two cylindrical plates made of polytetrafluoroethylene (PTFE) are mounted on both plates and are used to support the lenses. PTFE was chosen as the contact material for the lens surfaces. Because of its mechanical and inert properties, optical surfaces are protected during joining. In addition, two dial gauges were installed. To align and join the optical components, the two opposing plastic components are mounted between the PTFE plates. If both dial gauges indicate the same values, adhesive is applied to the adhesive gap provided. Loctite 406 (Henkel AG & Co. KGaA, Düsseldorf, Germany) was chosen as an adhesive because of its low viscosity and short curing time. Due to the low viscosity, the adhesive is drawn into the adhesive gap after application and distributed via capillary forces.

### 2.6. Process Monitoring

Process monitoring was performed throughout the process chain by the institute for applied optics (ITO). The ultra-precision machined master substrates were inspected in a Nanopositioning and nanomeasuring machine (NPMM-200) [29]. The written structures as well as the electroformed nickel structures were measured with a laser scanning microscope Mitaka MLP-3 (Mitaka Europe GmbH, Berlin, Germany) and a white light interferometer Veeco WykoNT9100 (Veeco Instruments, Inc., New York, NY, USA).

In order to test the optical function, a light beam was decoupled from a single-mode optical fiber and collimated by an achromat (AC. F = 200 mm), passed through the optical element, and was finally focused by an objective (Obj. f = 24 mm) onto a plane mirror. The fiber-coupled light source covers a wavelength range of 810 nm–870 nm. The laser beam is reflected back onto itself and coupled into a spectrometer via Y-coupler. The mirror is moved along the optical *z*-axis to determine the chromatic properties. By using this confocal approach, only the parts of the laser spectrum focused in the respective position are fed back and detected by the spectrometer.

## 3. Results

### 3.1. Ultra-Precision Turning and Laser Direct Writing of the Master Structures

Ultra-precision turning of the brass master structures was performed with a diamond tool from DiaTec GmbH with a tool radius of 0.705 mm. Since the surface quality is crucial for the following work steps, exact measurements were taken using a Mitaka MLP-3 surface profile measurement. The surface roughness was measured to be Ra = 0.013 µm.

**Figure 7:**
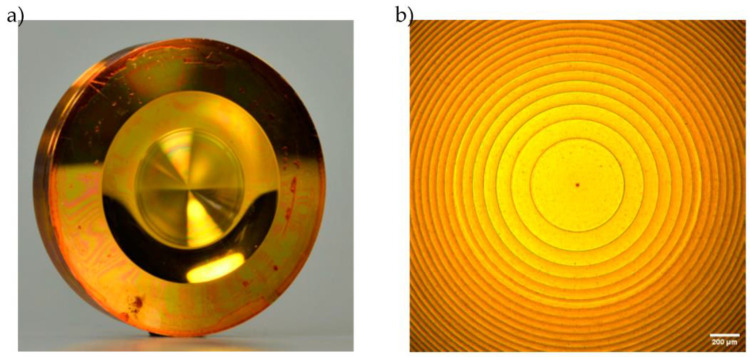
Brass substrate for electro plating. (**a**) Spin-coated brass substrate after laser direct writing. (**b**) Optical microscopy image of concentric optical surface structures on the substrate.

The photo curable resist was spin-coated onto the brass surface and resulted in a measured layer thickness of 2.1 µm. The high reflectivity of the brass surface was reduced by adding an additional 200 nm thick layer of anti-reflection coating (AZ^®^ BARLi^®^-II). The resist was structured via laser direct writing afterwards, resulting in concentric structures. The microstructured surface is shown in Figure 7.

### 3.2. Electroforming of the Substrate

A thin layer of gold (20 nm) was deposited on the brass master via physical vapor deposition (PVD) as a starting layer for the electroforming process. The brass master was coated with a nickel layer of about 4.5 mm. The nickel shim was cut via wire EDM to fit into the injection molding tool. For this purpose, the side walls of the nickel mold as well as the mounting plate were beveled in a 30° cone.

**Figure 8:**
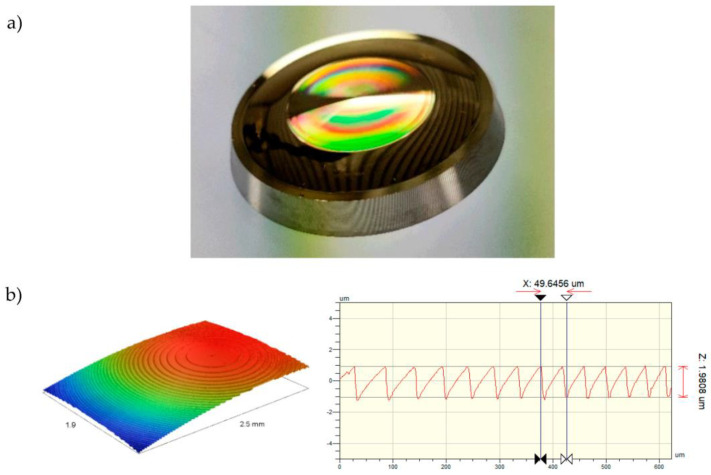
Electroformed nickel substrate machined for injection molding: (**a**) the molding insert with diffractive structures; (**b**) WLI measurement of the nickel substrates’ microstructured surface.

After integration of the mold insert, the polymer lenses were fabricated via injection compression molding. As shown in Figure 8b, the microstructures were successfully transferred onto the nickel surface.

### 3.3. Injection Compression Molding of Polymeric Optical Elements

The best results for injection molding were achieved with a tool temperature, including the mold insert being set to 80 °C. Compression forces were set to 20 kN. Additional molding parameters are presented in Table 4.

**Figure 9:**
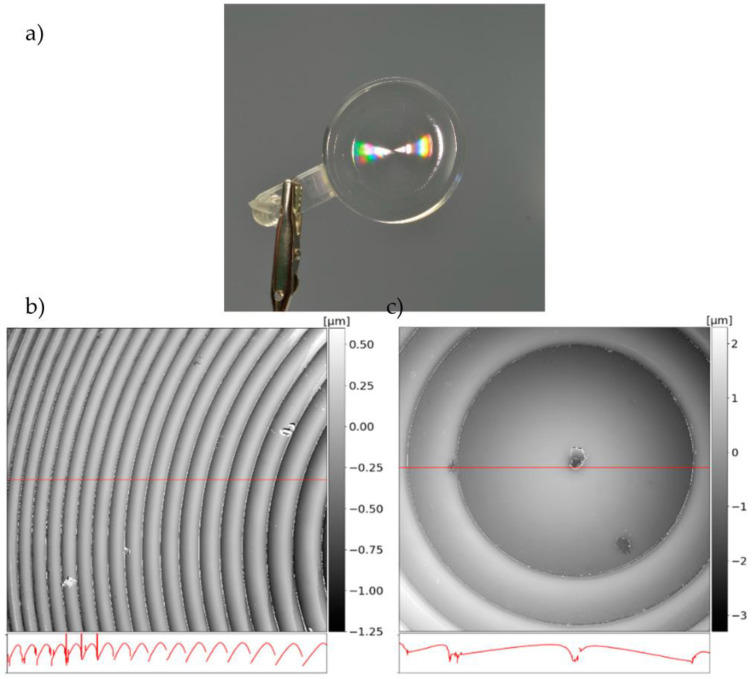
Injection-molded zero refractive element in PA6 Trogamid MyCX: (**a**) the diffractive structures were successfully transferred into the polymer surface as shown in (**b**); (**b**) NPMM measurement of the lens; (**c**) a small defect in the center of the lens was transferred into the polymer surface as well as the microstructures.

The rainbow effect indicates the diffraction of light when interacting with the microstructures. As seen in Figure 9b, the microstructures were successfully transferred onto the polymer surface. Smaller defects resulting from ultra-precision turning were transferred into the polymer as well as in the center of the lens structure shown in (c). Compared to the mold insert, the rounded edges of the structures suggest inconsistent filling behavior within the injection molding tool. A higher tool temperature in combination with vacuum-based venting could potentially generate sharper edges.

### 3.4. Optical Measurements

**Figure 10:**
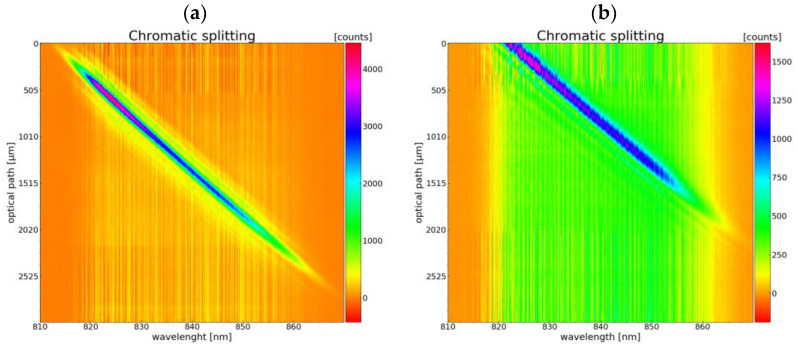
Chromatic longitudinal aberrations of (**a**) encapsulated zero refractive element—not glued and (**b**) glued zero refractive element; the adhesive process seems to have had a major impact on the signal distortion.

The zero refractive index element was conceptualized to be used in a chromatic confocal measurement setup. A demonstration setup was used to characterize the chromatic splitting of the element along the optical axis. The measurement results of the longitudinal chromatic aberrations are shown in Figure 10. The detected wavelength range of the spectrometer is plotted across the optical axis. The detected intensity is plotted as a function of the wavelength and the position of the mirror. It is shown that the detected intensity maximum shifts linearly to higher wavelengths with a change in the mirror position, thus showing the desired longitudinal chromatic aberration.

**Figure 11:**
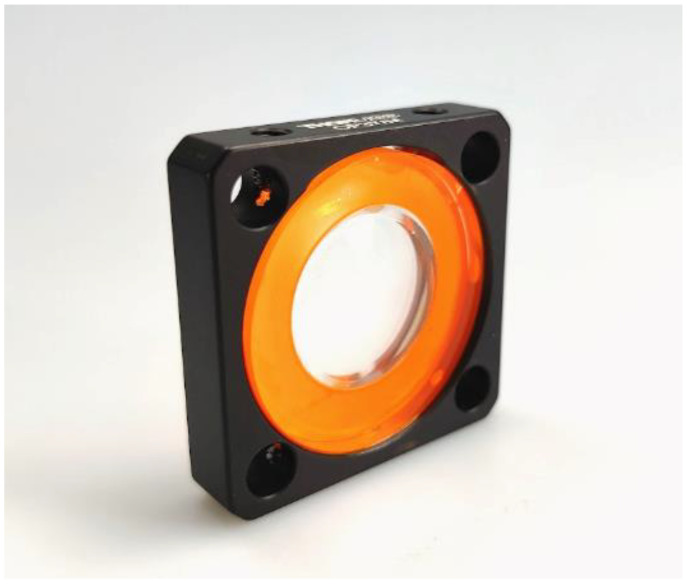
The orange-dyed adhesive spreads well on the surface provided for bonding, leaving out the microstructured surface in the middle of the lens.

The same setup was used to measure an assembled lens system containing the DOE and a matching polymer lens aligned and joined as described. The chromatic splitting signals produced by the glued DOE shown in Figure 10b demonstrate a shift in the zero position, which may be a deviation due to non-ideal bonding of the two lenses. Furthermore, the encapsulation of the surface microstructures was tested by mixing a color into the adhesive during the alignment process. As Figure 11 shows, no adhesive or liquid was drawn onto the microstructured surface but instead spread evenly on the alignment surface.

## 4. Discussion

The current article presents a method for manufacturing encapsulated optical components using injection compression molding. The manufacturing process involves the ultra-precise turning of brass masters, laser direct writing, and electroforming, followed by injection molding. A recent study suggested a process chain which involved the structuring of glass masters and electroforming [30]. A major disadvantage of this process chain was found in the relative adjustment of the laser structured glass masters to the final mold insert. Depending on the placement before electroforming, the resulting mold insert was misaligned and therefore did not fit the final position in the tool nor the polymer part. In the present process chain, the photo-sensitive polymer layer is microstructured directly on a precision-turned brass master. The master structure is afterwards electroformed and cut via wire electric discharge machining. We were able to reduce the adjustment mistakes in the injection molding tool to a minimum because the sufficient alignment of micro- and brass structures was achieved during the structuring process. Additionally, no mount for electroforming was needed because of the mechanical post-processing. Since the nickel mold thickness was more than 4 mm thick, simple mechanical deformation was possible. The most important step for the injection molding process turned out to be the following cleaning step, which included removing the remaining polymer in the nickel mold. The recommended AZ^®^100 cleaner proposed in this work proved to be highly effective for dissolving and removing the polymer residues, ensuring optimal conditions for demolding and a high accuracy during injection molding.

Overall, injection compression molding proved to be a powerful tool for the production of high-precision polymer lens systems. While the material PA6 Trogamid MyCX delivered good results in filling and demolding properties as well as in the optical properties, minor changes should be considered regarding the process design in the future. Rytka et al. state that vacuum-based injection molding becomes advantageous for feature sizes < 100 µm and inescapable for small feature sizes of less than a 1 µm [31]. In the future, a vacuum-based molding process should be taken in consideration. Another important influence, as stated by Roeder et al., is the mold temperature in regard to the filling behavior. They recommend molding with higher temperatures (up to 150 °C), as stated in the data sheet of the material [32] for PA6 Trogamid myCX, increasing the mold flow and the molding accuracy [15]. This has to be considered for future applications since higher temperatures affect the sticking behavior of the material in the tool and need to be monitored carefully in a mass production process.

Nevertheless, the process chain involves heavy-machinery-like diamond-based precision turning for the production of curved surfaces with low surface roughness, which is a standard process for optic manufacturers and delivers the highest surface quality, especially for aspherical lenses. The processing of diffractive structures has also been shown before, but it is often restricted in feature sizes due to the tool radius [33,34]. While the production of one DOE lens via ultra-precision milling is time- and cost-consuming, this study suggests a process chain to manufacture tools and inserts to produce multiple parts with the final tool. The electroplating process enables multiple copies of the master mold, relativizing the initial costs over time for large quantities. Once the tool is manufactured, multiple optical mold inserts can be used to produce different kinds of lenses with the same tool but with various microstructured inserts. Additionally, the quality of polymer optics based on laser direct writing is significantly higher than optics produced via ultra-precision turning, as shown in [30], leading to higher overall product quality. A further investigation of the process may lead to the development of polymeric lens systems with aspherical surfaces and diffractive structures, resulting in an imaging performance comparable to complex glass systems despite featuring only two microstructured polymer optics, making this process more suitable and cost-efficient than regular optics manufacturing.

## 5. Conclusions

In this study, a novel process chain to produce refractive polymer lenses with integrated diffractive microstructures on the surface is presented. The findings of this study can be summarized as follows:

- Overcoming the high initial costs results in the possibility of the flexible production of microstructured optics as different optical microstructures such as gratings, Fresnel structures, or DOEs can be transferred into the mold surface using laser direct writing followed by electro plating.
- The manufacturing of zero refractive elements using the suggested process chain has been proven successfully by transferring diffractive microstructures into the curved surface of refractive optics and encapsulating these via alignment structures integrated in the lens system.
- An initial approach for self-alignment during the adjustment of polymer lenses was presented as well. While the alignment structures integrated in the lens system were produced successfully in one step together with the lenses, the adjustment after production requires further investigation.

For future research, it is planned to validate the flexibility of the production process by introducing further integrated structures and also to use the advantage of ultra-precision milling to apply the process chain on aspherical lenses. Additionally, a further investigation into the adjustment and alignment process to produce complex encapsulated optical systems has to be conducted.

## Figures and Tables

**Table 1:** Turning parameters for the brass master with optical surface finish.

Parameter	Value
Feed rate [F]	3.0
Spindle speed [RPM]	2500
Infeed—roughing [µm]	0.003
Infeed—finish [µm]	0.001
Coolant	Isoparafin

**Table 2:** Spin coating parameters for the anti-reflection coating.

Parameter	Value
Spin time [s]	30
Acceleration [1/s^2^]	800
Spinning speed [RPS]	1000

**Table 3:** Spin coating parameters for the photoresist layer.

Parameter	Value
Spin time [s]	30
Acceleration [1/s^2^]	800
Spinning speed [RPS]	1500

**Table 4:** Injection molding parameters.

Parameter	Value
Melt temperature	290 °C
Mold temperature	
Fixed side	80 °C
Moveable side	80 °C
Compression force	20 kN
Cooling time	25 s
Holding pressure	100 bar

## Data Availability

Data presented in this study are available on request from the corresponding author.
